# Translational Research of Telecare for the Treatment of Hepatitis C

**DOI:** 10.1155/2014/195097

**Published:** 2014-06-10

**Authors:** Wan-Lin Chen, Wen-Ta Chiu, Ming-Shun Wu, Mei-Huang Hsu, Shin-Han Tsai

**Affiliations:** ^1^Institute of Injury Prevention and Control, Taipei Medical University, Taipei 110, Taiwan; ^2^College of Public Health and Nutrition, Taipei Medical University, Taipei 110, Taiwan; ^3^International SOS, Taipei 104, Taiwan; ^4^Ministry of Health and Welfare, Taipei 115, Taiwan; ^5^Division of Gastroenterology, Department of Internal Medicine, Wan Fang Hospital, Taipei Medical University, Taipei 116, Taiwan; ^6^Department of Emergency, College of Medicine, Shuang Ho Hospital, Taipei Medical University, Taipei 235, Taiwan

## Abstract

*Objective*. Chronic hepatitis C virus (HCV) infection is a serious health problem in Taiwan. The high dropout rate due to side effects limits the efficacy of treatment. The objective of this study is to investigate the effectiveness of telecare for the treatment of chronic hepatitis. *Material and Methods*. Two hundred and ninety-eight patients randomly chose either of the two support programs. Group 1 was offered public health nurse consultation at outpatient clinic. Group 2 was offered telecare program with 24 hours of consultation services via a health communication center. All patients were treated with standard therapy and followed up for 72 weeks. *Results*. Normalization of serum biochemistry was noted in both Group 1 (150 patients) and Group 2 (148 patients). The most common types of side effect in both groups were influenza-like symptoms. Patient compliance was 88% (Group 1) and 94.6% (Group 2). Total dropout cases were 18 (12%) in Group 1 and 8 (5.4%) in Group 2. The program costs were 232,632 USD (Group 1) and 112,500 USD (Group 2). *Conclusion*. Telecare system with health care communication center model is significant in reducing dropout rate and is more effective with easy access.

## 1. Introduction

Chronic hepatitis C virus (HCV) infection, including its sequelae, is an important healthcare problem in Taiwan. Nearly 5 million people, 22% of total population, are estimated to be infected and 15 to 20 % of the victims will develop cirrhosis and hepatocellular carcinoma (HCC) [1]. Current standard of treatment for patients with chronic hepatitis C is a 48-week combined therapy with pegylated interferon plus ribavirin. This combination regimen is associated with a sustained virologic response (SVR) in 38 to 43% of patients [2].

Since there is no hepatitis C vaccine available for primary prevention, health education is the most important control strategy for containing the impact of hepatitis C. Adherence to treatment regimens reduces viral load and complications while improving patient safety at home. However, side effects of treatment with interferon resulted in high dropout rates of about 26% in Taiwan and other countries [3, 4]. Therefore, improving adherence is a major concern in the management of hepatitis C patients.

Information and communication technologies (ICTs) have expanded access to patient care, increased efficiency, and reduced costs. Few of the types of information technology that may improve safety are widely implemented. In our previous studies, we have found that it is efficient and cost effective in emergency air medical service (EAMS) in remote islands, using a video-telemedicine system [5–7]. Multiple studies now demonstrate that computer-based decision support can improve physicians' performance and patient outcomes [8–10]. The burden of chronic disease can be significantly decreased through telemedicine-based programs. Therefore, a technology-enabled telecare program was provided in cooperation with treating physicians and educational support services.

The objective of this study is to investigate the effectiveness of telecare for the treatment of hepatitis C.

## 2. Methods

### 2.1. Patients

Patient selection criteria were defined as follows and are in accordance with the criteria of Fried et al. [4].

*Enrollment Criteria*.

1. adult patients who had never received interferon and who had at least 2000 copies of HCV RNA per milliliter of serum according to a polymerase-chain-reaction (PCR) assay (Cobas Amplicor HCV Monitor Test, version 2.0; Roche Diagnostics);
2. serum alanine aminotransferase activity above the upper limit of normal within six months before entry into the study;
3. a liver-biopsy result consistent with the diagnosis of chronic hepatitis C: serum HCV RNA levels above the linear range of the PCR (more than 1 million copies per milliliter) were diluted to within the linear range.

*Exclusion Criteria*.

1. neutropenia (<1500 neutrophils per cubic millimeter); thrombocytopenia (<90,000 platelets per cubic millimeter); anemia (<12 g of hemoglobin per deciliter in women and less than 13 g of hemoglobin per deciliter in men);
2. human immunodeficiency virus (HIV) infection;
3. decompensated liver disease;
4. a serum creatinine level more than 1.5 times the upper limit of normal;
5. poorly controlled psychiatric disease;
6. alcohol or drug dependence within one year before entry into the study;
7. substantial coexisting medical conditions.

### 2.2. Study Design

**Figure 1:**
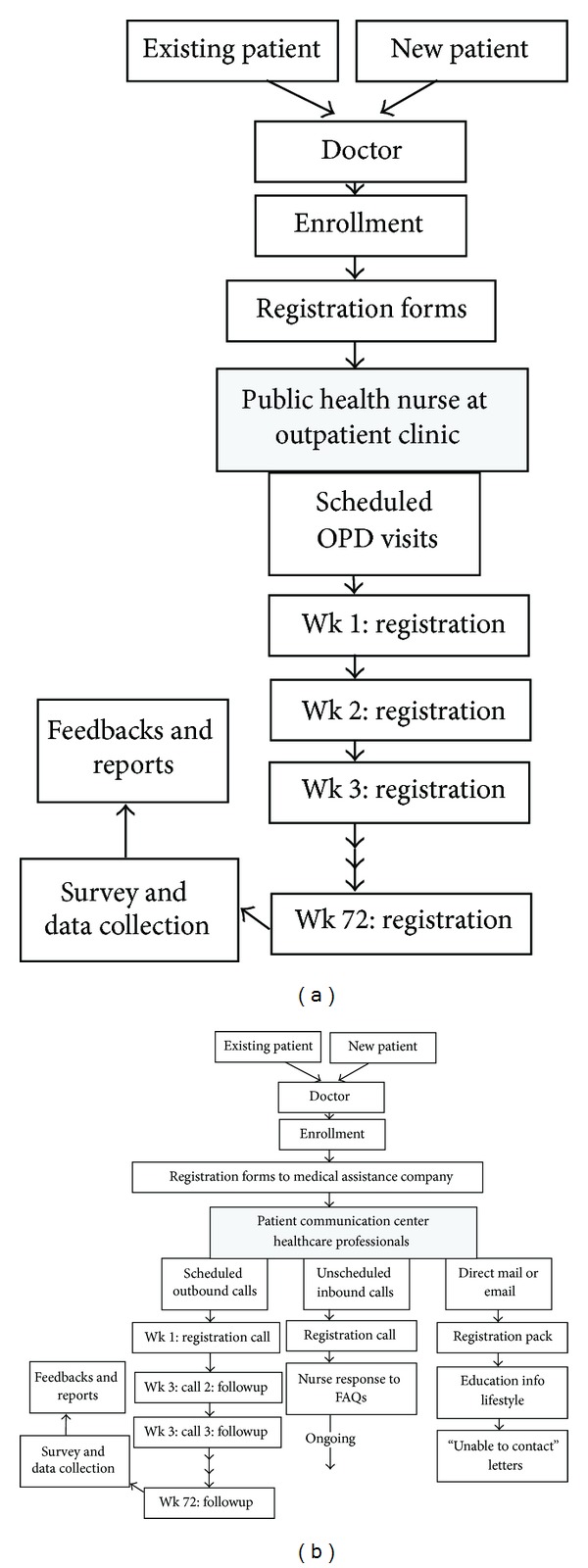
Standard operation procedures (SOPs) of support program in Group 1 (a) and telecare program in Group 2 (b).

All patients who met these criteria randomly chose to be serviced in either of the following two groups: (1) Group 1 was offered in-person public health nurse consultation in the outpatient clinic; (2) Group 2 was offered telephone consultation via a health communication center, whenever needed by the patient. Trained healthcare professionals including 4 nurses and 1 backup physician at the communication center made a series of structured, scheduled supportive phone calls to patients throughout their treatment period. The option of patient-initiated calls to the healthcare professionals at any time was available only to Group 2. Standard operation procedures of consultation and referrals in both groups are shown in Figure 1. Treatment plans in both groups were in accordance with treatment guidelines proposed by Asian Pacific Association for the Study of the Liver (APASL) [11].

All patients were treated with standard therapy, which included subcutaneous injection with peginterferon alfa-2a (PEGASYS, Hoffman-La Roche, NJ) 180 *μ*g once weekly and oral ribavirin (ROBATROL, Hoffman-La Roche, NJ) 800 to 1200 mg daily for 48 weeks. Patients were followed up until week 72 to assess if there was a sustained virologic response (SVR) to treatment.

### 2.3. Cost

The expenses of the medical treatment were all paid by the government in both groups. The patients did not have to pay any fee for the medications and support programs. Both support programs were funded by the National Health Research Institute and Department of Health, Taiwan, in this study. The public health nurses in Group 1 or healthcare professionals in Group 2 were paid with a fixed salary and would not receive any bonus or rewards related to the results of this study.

### 2.4. Assessment of Efficacy

The primary outcome measure was SVR, defined as the absence of detectable HCV RNA at the end of followup with a PCR assay. By week 12, 86% of patients treated with peginterferon alfa-2a plus ribavirin had had a virologic response, defined as a 2-log decrease from baseline HCV RNA levels or no detectable serum HCV RNA. The absence of an early virologic response was not associated with early treatment discontinuation (before week 12) or dose modification. Of those with early virologic responses, 65% subsequently had a SVR. Those with no detectable HCV RNA by week 12 were more likely to have a SVR than those who had only a 2-log decrease in HCV RNA. In contrast, among patients who did not have an early virologic response, 97% did not have a SVR [4]. For patients with at least 20 weeks of followup, the last observed HCV RNA level was used in assessments of efficacy. Patients with followup of less than 20 weeks were considered to have no response to treatment.

### 2.5. Assessment of Patient Safety and Adherence

The secondary outcome was defined as safety and dropout rate. Safety was assessed by laboratory tests and evaluation of adverse events at weeks 1, 2, 4, 6, and 8; monthly thereafter during treatment; and at weeks 52, 60, and 72. Patients who discontinued treatment prematurely because of intolerance were encouraged to remain in the study. The treating doctor would consider dose reduction stepwise to let patients remain in the study. Stepwise reductions in the peginterferon alfa-2a dosages to 135, 90, or 45 µg per week and reductions in ribavirin dosages to 800 or 600 mg per day were allowed to alleviate adverse events or laboratory abnormalities. If the adverse events resolved, a return to initial dosing levels was permitted. Patient adherence was determined by the number of patients who received complete treatment regimen and posttreatment followup. Dropout rate was measured by the number of patients who discontinued treatment prematurely or lost to followup. We further classified dropout types into physician-driven and patient-driven reasons. The decision of dropout is in the discretion of either treating physician or patient. Every single adverse event was considered an independent event to assess the percentage of all kinds of side effect.

### 2.6. Data Collection

Data collection included two abstractors who received training on the use of computer software “SPSS 11.0.” The abstractors did a detailed review of the data elements and definitions. The database was reviewed for validity and reliability as well. All data in both groups including patient demographics, laboratory data, adverse events, and costs were entered into SPSS for coding and statistical analyses. The research associate in charge of the study developed the data codebook, and the abstractors were blinded to the research questions being tested. Cronbach's *α* method was applied in the analysis of reliability. We also used confirmatory factor analysis method to evaluate validity.

### 2.7. Statistical Analysis

The results are evaluated by frequency in each group. Continuous variables in the two groups are compared by Student *t*-test. All *P* values were two tailed, and a *P* value < 0.05 was considered statistically significant.

## 3. Results

### 3.1. Patient Demographics

A cohort of 150 and 148 patients was selected from 12 hospitals throughout Taiwan in Group 1 and Group 2, respectively. Mean age of patients in the two groups was 51.5 and 46.5 years. There were no statistically significant differences in the demographics between the two groups. The basic data of patients (age, sex, genotype, and pretreatment lab data) are summarized in Table 1.

### 3.2. Biochemistry and Virologic Response

Normalization of mean posttreatment serum aspartate aminotransferase (AST) andalanine aminotransferase (ALT) was noted in both Group 1 and Group 2. SVR rates of 66% and 68.9% were noted in the patients in Group 1 and Group 2, respectively (Table 2). Among patients with genotype 1, high baseline viral RNA levels (more than 2 million copies per milliliter) were noted.

### 3.3. Patient Safety

The most common types of side effect in both groups were influenza-like symptoms (26.0% and 28.6%). Other common side effects included gastrointestinal distress and psychiatric symptoms. The most common causes leading to discontinuation were depression-related events in both groups. One death occurred during the treatment period in Group 1 (Table 3).

A total of 132 and 140 patients completed the course of treatment and followup in Group 1 and Group 2, respectively. Patient compliance was 88% and 94.6%, respectively. Total dropout cases in the Group 1 and Group 2 were 18 and 8, respectively. Of the 18 dropout cases in Group 1, 4 patients (22.2%) discontinued therapy by treating physicians because of intolerable side effects and 14 (77.8%) discontinued therapy by themselves. In Group 2, there were 7 (87.5%) physician-driven dropouts and 1 case (12.5%) of patient self-dropout. Total dropout rates were 12% and 5.4% in Group 1 and Group 2, respectively (*P* < 0.05, Table 4).

### 3.4. Cost Analysis

Cost analysis is based on the cost of the programs for treatment and follow-up period (72 weeks) in the two groups. In Group 1, the monthly pay of each public health nurse was 35,000 NTD (US$ 1,077). This study collected data from 12 hospitals nationwide. Therefore, the cost of the program in Group 1 is 232,632 ($1,077 × 18 × 12) dollars. In Group 2, the annual budget of support program, including operating system and the nurse, was US$75,000. The nurse spent all the funded time to the study patients. Thus, the cost of the program in Group 2 is 112,500 ($75,000 × 18/12) dollars (Table 2).

## 4. Discussion

HCV infection is one of the major health problems in Taiwan. Among patients with non-A, non-B fulminant hepatitis, antibodies against HCV (anti-HCV) or serum HCV RNA were found in 40 to 60 percent in Taiwan. There was only 2 percent (range, 0 to 12 percent) in Western countries, with one exception: a recent study conducted in California reported a prevalence of 60 percent associated with low socioeconomic status and Hispanic ethnicity [12]. The prevention of hepatitis and control of the sequelae pose a great challenge to the health care system.

In patients with HCV infection, improvements in combination therapy have resulted in higher response rates. Our study showed that peginterferon alfa-2a and ribavirin produce high rates of virologic and biochemical responses and the findings are comparable to previous series with combination therapy [1, 2, 13]. Treatment protocols for HCV infection are difficult to endure and require a serious commitment on the part of the patient. Adherence to treatment regimens has been associated with the side effects of medications. In the previous series, overall compliance is 74%. One-fourth of the patients withdrew from treatment due to treatment-related adverse events [4]. In the present study, the compliance rates in both Group 1 (88%) and Group 2 (94.6%) are higher than those without patient support program, which has been well studied in Taiwan and in other countries [3, 4, 14, 15].

Studies show that race, sex, and socioeconomic status have not been consistently associated with levels of adherence [9]. Effective strategies for improving adherence range from basic clinical practices—such as establishing a consistent, trusting relationship with the patient, providing clear information about the intended effects and possible side effects of medication, and paying careful attention to perceived side effects—to special strategies such as reminder systems. The purpose of this study is to find an effective strategy with random selection of two kinds of program in the same clinical settings for populations of similar race, sex, and socioeconomic status. Even though patients in the two groups had similar types and severities of adverse events, with similar numbers of episodes of anemia and leucopenia (*P* > 0.05), we found that the percentage of patients who discontinued medical treatment in Group 2 is significantly less than that in Group 1 (*P* < 0.05).

There is increasing evidence that high-quality and cost-effective health interventions improve medication compliance and quality of life [8, 9, 16–18]. The advances in development of ICTs provide a key supplemental strategy to enhance adherence. ICTs can reduce adverse events by facilitating a more rapid response after an adverse event has occurred and tracking and providing feedbacks [19–21]. Comparing the reasons of discontinuation, patient self-dropout was the major (77.8%) cause in Group 1, while in Group 2, patient self-dropout only comprises the minority (12.5%) (Table 4). The significant difference in the percentage of patient self-dropout demonstrates the supportive functions of the telecare program in Group 2. With the assistance of ICTs, the treating physicians were more alert to any adverse effects during the course of treatment and followup and the patient-physician relationship may be enhanced. In the present study, the built-in pop-up windows in Group 2 not only monitor patient adherence but also remind healthcare professionals to contact the physicians before serious complication occurs. This unique program shifts the emphasis from episodic care of the traditional care to continuous care [22]. The patients in the telecare consultation group are more feasible because they can call anytime. However, in person consultation might have certain fixed timings which is inconvenient for patients to get. Moreover, the personnel in the telecare program move from the role of medical authority to that of medical facilitator, guiding patients through a patient-centered model of care. This integration of ICTs allows for greater access to medical expertise. Providing reliable, efficient individualized patient support requires a degree of mastery of data and coordination with increased use of ICTs. When compared with the cost of the support program in the two groups, the cost in Group 2 is only half of Group 1. The present study demonstrates that the compliance of the patients from the same treating physicians and hospitals is significantly different between Group 1 and Group 2. Moreover, the cost of telecare in Group 2 is significantly less than that in Group 1. The success of the program in Group 2 is based on a combination of a well-structured and patient-centered program design, well-trained professional nurses, and friendly protocols that efficiently manage patient cases on a personalized basis.

This is the first study to evaluate the effectiveness of telecare in chronic hepatitis C treatment. The present study demonstrates positive effects in the following aspects. It suggests new treatment protocols with an effective telecare program in the management of hepatitis C. It helps patients to overcome the challenges and encourages a higher percentage of patients' adherence to their treatment plan with the patient-centered approach. Subsequently, it achieves better clinical outcomes. It also provides patient-friendly program and may improve patient-physician relationship. Furthermore, it improves patient safety with the adoption of ICTs. Incorporations of the telecare for the treatment of other chronic diseases, such as hypertension, epilepsy, and anxiety disorder, are under investigation. This study highlights the important role of ICTs in patient support program.

In conclusion, as burden related to hepatitis and other chronic diseases increases, we suggest a cost-effective intervention, technology-based and patient-centered telecare program, which may reduce dropout rate, improve the outcome, help patient safety, and save the budget of public health in the treatment of chronic hepatitis C.

## Figures and Tables

**Table 1:** Patient demographics in the two groups.

Number of patients	150	148
Mean age (yr)	51.5	46.5
Genotype		
1	87 (58)	90 (60.8)
2	35 (23.3)	31 (20.9)
3	20 (13.3)	21 (14.2)
4	8 (5.4)	6 (4.1)
Mean pre-Tx AST (U/liter)	172.1	166.3
Mean pre-Tx ALT (U/liter)	360.8	380.9
Mean pre-Tx HCV RNA (number of copies/mL ∗ 10^−6^)	369	381

AST: aspartate aminotransferase.

ALT: alanine aminotransferase.

Tx: treatment.

**Table 2:** Effectiveness in both groups.

	Group 1 (*n* = 150)	Group 2 (*n* = 148)
Mean post-Tx AST (U/liter)	59.7	62.1
Mean post-Tx ALT (U/liter)	51.3	56.2
Mean post-Tx HCV RNA (number of copies/mL ∗ 10^−6^)	0.19	0.23
Number of patients with SVR (%)	99 (66)	102 (68.9)
Cost (USD)	232,632	112,500

**Table 3:** Comparison of patient safety profiles in the two groups.

	Group 1 (*n* = 150)	Group 2 (*n* = 148)
	Number of patients (%)	
Any adverse events	497	504
Influenza-like symptoms	129 (26.0)	144 (28.6)
Gastrointestinal symptoms	96 (19.3)	111 (22.0)
Psychiatric symptoms	75 (15.1)	84 (16.7)
Anemia	59 (11.9)	52 (10.3)
Dermatologic symptoms	49 (9.9)	51 (10.1)
Leucopenia	48 (9.7)	47 (9.3)
Thrombocytopenia	41 (8.1)	15 (3.0)
Death	1	0

**Table 4:** Comparison of dropout rate and compliance.

	Group 1 (*n* = 150)	Group 2 (*n* = 148)
Dropout*		
Physician-driven*	4 (22.2%)	7 (87.5%)
Patient self-dropout*	14 (77.8%)	1 (12.5%)
Total (%)	**18 (100%)**	**8 (100%)**
Dropout rate* (%)	12	5.4
Compliance rate* (%)	88	94.6

*P < 0.05.
